# Qiweibaizhu Decoction Treats Diarrheal Juvenile Rats by Modulating the Gut Microbiota, Short-Chain Fatty Acids, and the Mucus Barrier

**DOI:** 10.1155/2021/8873294

**Published:** 2021-01-17

**Authors:** Shaodan Sun, Yang Yang, Xiaojie Lin, Peiwen Chen, Liyan Ye, Liying Zeng, Qina Ye, Xiangna Yang, Jingtu Ceng, Jiayi Shan, Li Xie, Meirong Jiang, Fei Luo, Xiaogang Chen

**Affiliations:** ^1^The Second Clinical College of Guangzhou University of Chinese Medicine, Guangzhou University of Chinese Medicine, Guangzhou 510120, China; ^2^Lingnan Medical Research Center, Guangzhou University of Chinese Medicine, Guangzhou 510407, China; ^3^Affiliated Jiangmen Traditional Chinese Medicine Hospital of Ji'nan University, Ji'nan University, Jiangmen 529000, China; ^4^Integrated Chinese and Western Medicine Postdoctoral Research Station, Jinan University, Guangzhou 510632, China; ^5^Guangdong Provincial Hospital of Traditional Chinese Medicine, Guangzhou 510120, China; ^6^Department of Pediatrics, The First Affiliated Hospital of Guangzhou University of Chinese Medicine, Guangzhou 510405, China; ^7^Guangzhou Institute of Pediatrics, Guangzhou Women and Children's Medical Center, Guangzhou 510407, China; ^8^Tongde Hospital of Zhejiang Province, Hangzhou 310012, China; ^9^Guangzhou University of Chinese Medicine, Guangzhou 510405, China

## Abstract

**Background:**

Qiweibaizhu decoction (QBD), a classic Chinese herbal formula, has been widely used for treating diarrhea in infants and children with spleen deficiency syndrome for centuries, but its mechanism of action remains unclear. The gut microbiota, short-chain fatty acids (SCFAs), and intestinal mucus are closely associated with diarrhea.

**Methods:**

In this study, the composition of the gut microbiota in diarrheal rats was analyzed by 16S rDNA amplicon sequencing. The concentrations of colon SCFAs were determined using gas chromatography-mass spectrometry (GC-MS). The expression of mucin 2 (MUC2) in the colon was detected by immunofluorescence.

**Results:**

Diarrhea significantly changed the diversity and structure of the gut microbiota and disrupted the mucus barrier in juvenile rats. QBD did not significantly change the diversity and structure of the intestinal flora, but it enhanced the increasing tendencies of Verrucomicrobia and *Akkermansia* and decreased the abundance of *Turicibacter* (*P*=0.037) and *Flavonifractor* (*P*=0.043). QBD tends to repair the mucus layer and promote MUC2 expression in juvenile rats with diarrhea. Moreover, *S. boulardii* significantly increased the abundance of *Parasutterella* (*P*=0.043). In addition, QBD treatment tends to increase the propionic acid concentration during diarrhea, but its levels of acetic acid, propionic acid, butyric acid, and total SCFAs were lower than those in the *S. boulardii* group.

**Conclusion:**

*S. boulardii* significantly increased the abundance of *Parasutterella*, leading to increased production of acetic acid, propionic acid, and butyric acid, consequently leading to alleviation of diarrhea. In comparison, QBD affected diarrhea via regulation of the intestinal flora, especially by increasing the abundance of Verrucomicrobia and *Akkermansia,* resulting in mucus barrier repair, protection of the intestines, and treatment of diarrhea.

## 1. Introduction

Diarrhea was the eighth leading cause of death among people of all ages and the fifth leading cause of death among children under 5 years of age in 2016, especially in South Asia and sub-Saharan Africa [1, 2]. Osmotic diarrhea is a common disease in humans caused by food intolerances, malabsorption, and the widespread use of laxatives [3]. Polyethylene glycol (PEG) is a commonly used digestive remedy to induce osmotic diarrhea. Osmotic diarrhea can significantly change the microbial community structure, which is similar to changes observed in other gastrointestinal diseases, including inflammatory bowel disease (IBD) [4].

Diarrheal disease is associated with dysbiosis of the gut microbiota in children [5]. The Global Enteric Multicenter Study found that moderate-to-severe diarrhea reduced bacterial diversity and altered microbiota composition in children [6]. Children with multiple episodes of diarrhea in early life may suffer impairment in gut microbiota development that could result in persistent diarrhea, malnutrition, and immune system diseases. Pre- and/or probiotics can modify the microbiota and improve the outcomes [7, 8].

Short-chain fatty acids (SCFAs), mainly produced by various gut microflora in the colon, are fatty acids with carbon chains containing less than 8 carbon atoms, mainly including acetic acid, propionic acid, and butyric acid [9]. SCFAs are an important energy for intestinal cells and are key signaling molecules for maintaining intestinal health [10]. SCFAs are absorbed by colonic epithelial cells and stimulate Na-dependent water and electrolyte absorption to alleviate diarrhea symptoms [11]. Specifically, sodium butyrate can also prevent diarrhea by increasing the passive absorption of water by the colon and affecting the intestinal microflora [12].

Mucin is a large, highly glycosylated protein that is important for protecting the lumen of the gastrointestinal tract [13].Recently, a large number of studies have shown that the mucus barrier and its most important protein, mucin 2 (MUC2), protect the intestines and prevent diarrhea. Tropini et al. confirmed that diarrhea is closely related to the gut microbiota and the intestinal mucus barrier. Diarrhea significantly changes the gut microbiota and makes the mucus layer thin or absent [3]. Changes in the composition of the intestinal flora and the balance of the environment in the intestine can lead to a decrease in MUC2 secretion [14].

Probiotics in the pediatric population have been widely studied in the prevention and treatment of diarrheal diseases [15]. *S. boulardii* is a nonpathogenic probiotic yeast that is tolerated with gastric acid and antibiotics [16]. *S. boulardii* has been reported to reduce signs of diarrhea related to or caused by various factors in animals and humans [17, 18]. The mechanisms by which *S. boulardii* treats diarrhea are as follows: (1) *S. boulardii* can inhibit the growth and invasion of pathogens [19]. (2) *S. boulardii* can inhibit host cell adherence that interferes with bacterial colonization [20]. (3) *S. boulardii* has an antisecretory effect [21]. (4) Importantly, *S. boulardii* was reported to reduce bacterial gut translocation and improve intestinal barrier function in animal models [22]. *S. boulardii* can affect the composition and release of intestinal mucin, enhance the mucin barrier, and reduce the penetration of SN-38 into epithelial cells, thereby reducing mucosal damage [23]. At present, the mechanism of action of *S. boulardii* is not yet fully understood, and the optimal dose of its action remains to be explored.

In traditional Chinese medicine (TCM), combination therapies (also called formulae) have been used for 2,500 years. Formulae are a combination of several types of medicinal herbs or minerals, which aim to enhance therapeutic efficacy and reduce adverse effects [24]. The Qiweibaizhu decoction (QBD), consisting of Guanghuoxiang (*Pogostemon cablin* (Blanco) Benth.), Gegeng (Radix Puerariae), Baizhu (*Atractylodes macrocephala* Koidz.), Rensheng (*Panax ginseng* C. A. Mey.), Fuling (*Poria cocos*(Schw.) Wolf.), Muxiang (Aucklandiae Radix), and Gancao (Licorice), has been used to treat the diarrhea associated with spleen deficiency syndrome since the Song Dynasty (960–1127 AD). The term “spleen” in traditional Chinese medicine (TCM) is different from that in Western medicine. Spleen deficiency syndrome is characterized by poor appetite, fullness, fatigue, pale tongue coating, weight loss, and loose stools. The commonly used treatment duration of QBD is 3–14 days. The commonly used dose range of QBD is 0.64–2.56 g/ml; 0.64 g/ml is a low dose, 2.56 g/ml is a high dose, and the most commonly used dose is 1.6 g/ml [25–28]. In these safe ranges, no obvious side effects or adverse reactions have been reported [29–33]. In this experiment, the dose of QBD was 1.20 g/ml for 10 days, which is a low dose compared with other experiments. Moreover, the seven traditional Chinese medicines in QBD are very safe traditional Chinese herbs, which can be eaten as food. In traditional Chinese medicine, these are called “drug homologous food.”

A number of studies have confirmed that QBD can balance the gut microbiota by promoting the growth of beneficial bacteria and inhibiting the reproduction of harmful bacteria [34]. The therapeutic effect of QBD depends on its various effective chemical components, such as polysaccharides, saponins, proteins, daidzein, and flavonoids, which are the material basis for the therapeutic effects of QBD [35–40]. Another study found that QBD contains a small amount of prebiotic substances; these compounds can promote the growth of probiotics in the intestine and can promote the synthesis of a variety of proteins and vitamins in the intestine [41]. Moreover, Sun et al. found that QBD is effective for treating diarrhea in mice by regulating the imbalance of intestinal flora and promoting the repair of intestinal mucosal damage [32]. Zhang et al. showed that the four herbs in QBD can increase the expression of MUC2 to improve the state of spleen deficiency in rats [62].

Therefore, we hypothesized that QBD could regulate the structure of the gut microbiota and the levels of SCFA and MUC2 in the treatment of diarrhea. Specifically, we applied QBD to treat juvenile rats with diarrhea and compared the results with those obtained with *S. boulardii* to explore whether QBD treats diarrhea by modulating the gut microbiota and SCFA-producing bacteria to increase the concentrations of SCFAs or MUC2-producing bacteria to increase the expression of MUC2.

## 2. Methods

### 2.1. Identification of Bioactive Ingredients of QBD

The Traditional Chinese Medicine System Pharmacology Database and Analysis Platform (TCMSP) (http://tcmspw.com/tcmsp.php) is a systems pharmacology platform for Chinese medicine. By using the TCMSP, the chemical components of each Chinese medicine compound in QBD can be obtained, and the bioactive ingredients can be screened according to the absorption, distribution, metabolism, and excretion (ADME) information of the Chinese medicine components. The screening criteria are as follows: OB value (oral bioavailability) ≥30%, OB% ≥60% for (Gancao) licorice, and DL value (drug-likeness) ≥0.18 [42].

### 2.2. Preparation of QBD

QBD was prepared by combining seven herbs in a ratio of 10 : 10 : 10 : 10 : 5 : 4 : 2 by weight of Guanghuoxiang (*Pogostemon cablin* (Blanco) Benth.), Gegeng (Radix Puerariae), Baizhu (*Atractylodes macrocephala* Koidz.), Rensheng (*Panax ginseng* C. A. Mey.), Fuling (*Poria cocos*(Schw.) Wolf.), Muxiang (Aucklandiae Radix), and Gancao (Licorice). These herbs were obtained from Guangdong Provincial Hospital of Traditional Chinese Medicine. These herbal materials were soaked in 4 times the volume of cold water in a beaker for 30 min and decocted 2 times (45 min each time). Rensheng (*Panax ginseng* C. A. Mey.) was soaked and decocted separately. The above mixture was thus obtained and concentrated to a concentration of 1.20 g of the original medicinal material per milliliter of the medicinal liquid by using a rotary evaporator in an 80°C water bath. After the decoction had cooled, it was stored in a freezer at −20°C until use.

### 2.3. *S. boulardii* Preparation

*S. boulardii* (Ultra-Levure® from Biocodex) was suspended in distilled water and administered orally. Each bag contained 765 mg of powder and 250 mg of bacterial powder; the number of viable bacteria per 1 g of powder should not be less than 1.3 × 10^9^ CFU. A solution (12 × 10^10^ CFU/kg) was prepared with pure water.

### 2.4. Experimental Animals and Modeling

Forty-eight specific-pathogen-free male Sprague-Dawley rats (age, 5 weeks; weight, 142 ± 8 g) were purchased from the Guangdong Animal Experimental Center (license no. SCXK (Yue) 2018–0002). The rats were kept in standard cages at the Experimental Animal Center of the First Affiliated Hospital of Guangzhou University of Chinese Medicine (license no. SYXK (Yue) 2013–0092) under standard environmental conditions (21–25°C; 50%–60% humidity; 12 h light/dark cycle). Diet and water were freely available throughout the experiment. All experiments involving animals were approved by the Animal Experimentation Ethics Committee of the First Affiliated Hospital of Guangzhou University of Chinese Medicine (approved application no. TCMF1-2019036) and were in compliance with the Guidelines for the Care and Use of Laboratory Animals published by the US National Institutes of Health. All efforts were made to minimize the suffering of the animals.

### 2.5. Experimental Processes

The rats were randomly assigned to 4 groups (*n* = 12): control, model control, *S. boulardii*, and QBD groups. The control group did not receive any treatment; the model group, *S. boulardii* group, and QBD group were treated with polyethylene glycol (PEG) water (15% PEG 3350 was added to the drinking water for the first three days, and 10% PEG 3350 was added for the remaining six days) and were placed on a small platform in a water environment to stand for 6 hours for 9 days.

After the nine days and 12 hours of PEG induction, rats in the model, *S. boulardii*, and QBD groups were intragastrically given normal saline (1 ml/kg), *S. boulardii* (1 ml/kg), and QBD (1 ml/kg), respectively, once a day for 10 days. Defecation, body weight, diet, water, and general clinical conditions were monitored every day.

Colon contents were collected and immediately frozen with liquid nitrogen after sampling and stored at −80°C. Tissue samples from the colon were immediately fixed in methanol-Carnoy's solution (60% dry methanol, 30% chloroform, and 10% glacial acetic acid).

### 2.6. Diarrhea Assessment

Stool consistency was classified according to the following visual grading scale as previously described [43]: (1) formed, stool maintains its shape, brown, score = 1; (2) semiformed or soft, does not pour, yellow, score = 2; and (3) liquid, pours more easily, yellow, score = 3.

### 2.7. Analysis of 16S rDNA in the Colon Contents

#### 2.7.1. Sample Collection and DNA Extraction

The composition of the gut microbiota was detected by 16S rDNA amplicon sequencing analysis. Total DNA was isolated from the colon content samples with the DNA Stool Mini Kit (Qiagen, Hilden, Germany) according to the manufacturer's protocols. DNA concentration and purity were determined by spectrophotometry (ND-2000 spectrophotometer, Thermo Fisher Science, Waltham, MA, USA). DNA integrity and concentration were assessed using 1% agarose gel electrophoresis.

#### 2.7.2. 16S rRNA Gene Amplification, Sequencing, and Illumina MiSeq

The V3-V4 region of the bacterial 16S rDNA was amplified by PCR (95°C for 5 min; 25–28 cycles of 98°C for 20 s, 58°C for 15 s, and 72°C for 20 s; and 72°C for 5 min, followed by holding at 4°C). The amplicon was detected using 2% agarose gel electrophoresis and purified by the AxyPrep DNA Gel Extraction Kit (Axygen Biosciences, Union City, CA, USA). The amplified products were generated using the Illumina MiSeq system (Illumina, San Diego, CA, USA) with barcoded primers. The amplicons were sequenced (PE250) on an Illumina MiSeq platform (Shanghai Realbio Institute, Shanghai, China) to obtain 250 bp paired-end reads.

#### 2.7.3. Sequencing Data Analysis

Paired-end sequencing was performed, and ^*∗*^_1.fq and ^*∗*^_2.fq each correspond to a FASTQ file. Paired-end sequencing was performed on the Illumina platform. The reads were spliced based on the overlap between reads, and quality control of the spliced reads was performed to obtain clean reads. The optimized clean reads were obtained by Pandaseq, and an in-house program was used to process the spliced reads as follows to obtain clean reads: (1) reads with an average quality value of less than 20 were removed; (2) reads with more than 3 N bases were removed; and (3) reads not in the length range of 220∼500 nt were removed. The operational taxonomic units (OTUs) for species classification were obtained by using usearch for clustering with 0.97 similarity, and chimeric filtering was performed on the clustered sequences.

### 2.8. Determination of SCFAs in the Colon Contents

#### 2.8.1. Sample Preparation

Colon contents (50 mg) were weighed. Saturated sodium chloride solution (400 *μ*L) and saturated sodium chloride solution with hydrochloric acid (50 *μ*L, 3 mmol) were added, and the combination was mixed by ultrasonication for 1 hour at low temperature. Cold ether (500 *μ*L) was then added to the combination. The mixture was then centrifuged at 12000 r/min and 4°C for 10 min. The supernatant was removed and collected into a tube containing 0.1 g of anhydrous sodium sulfate and vortexed for 3 min. Finally, the supernatant was obtained by centrifugation (4500 r/min and 4°C for 5 min) for determination of the SCFAs by gas chromatography-mass spectrometry (GC-MS).

#### 2.8.2. Instrumental Conditions

The instrument used in this experiment was a GC-MS Agilent 7890B gas chromatograph and an Agilent 5977A MSD mass spectrometer in series for detection in selected ion monitoring (SIM) mode. MSD ChemStation software was used for data processing to calculate the absolute content of the target compound in the sample. (1) The required gas chromatographic parameters were as follows: chromatographic column: Agilent HP-FFAP (25 m + 0.32 *μ*m + 0.50 mm); injection volume: 2 *μ*L; split ratio: 20 : 1; injection port: 250°C; flow rate: 1.5 mL/min; heating program: the oven program had an initial temperature of 100°C, which was increased to 150°C at a rate of 5°C/min, and maintained at 230°C for 2 min. (2) The mass spectrometry parameters were as follows: ion source: EI+; ion source temperature: 280°C; transmission line temperature: 250°C; solvent delay: 3.5 min; scanning range: scan 35–200 m/z. The SIM mode was chosen to determine the ion mass of each SCFA, including m/z values of 41, 43, 45, 57, 60, 73, 74, 87, and 88.

### 2.9. Alcian Blue Staining

Tissue samples from the colon were immediately fixed in methanol-Carnoy's solution (60% dry methanol, 30% chloroform, and 10% glacial acetic acid) and processed in paraffin as previously described [44, 45]. Sections were washed in xylene I for 20 min, xylene II for 20 min, anhydrous ethanol I for 5 min, anhydrous ethanol II for 5 min, 75% alcohol for 5 min, and tap water for 5 min. Sections were stained with Alcian blue dye for 15 min and then washed with tap water. Finally, sections were dehydrated with anhydrous ethanol I (5 min), anhydrous ethanol II (5 min), anhydrous ethanol III (5 min), xylene I (5 min), and xylene II (5 min).

### 2.10. Immunofluorescence

Tissue samples from the colon were fixed and processed as described for Alcian blue staining. Sections were washed in xylene I for 15 min, xylene II for 15 min, anhydrous ethanol I for 5 min, anhydrous ethanol II for 5 min, 85% alcohol for 5 min, 75% alcohol for 5 min, and distilled water for 5 min. Sections were immersed in citric acid antigen retrieval solution and boiled in a microwave oven (300 W) for 5 min twice. Then, the sections were soaked in hot solution for 20 min. Sections were washed in PBS 3 times (each time for 5 min) and marked with a PAP (liquid blocker) pen. BSA was added, and the samples were incubated in the dark in a humid chamber for 30 min at room temperature. A specific antibody for MUC2 (Servicebio, diluted 1 : 200 in blocking solution) was added to the slide, which was then incubated in darkness for 4–24 hours at 4°C. The slides were washed three times in PBS. The secondary antibody (Servicebio, diluted 1 : 200 in blocking solution) was added to the sample, which was then incubated in the dark at room temperature for 50 min. After incubation with the secondary antibody, slides were washed three times in PBS and dried. DAPI (Servicebio) was then added to the slides for incubation in the dark at room temperature for 10 min. Finally, the slides were washed three times in PBS and mounted with Antifade Mounting Medium (Servicebio). Slides were stored at 4°C in the dark until imaging.

## 3. Data Analysis

Statistical analysis was performed with STATA 12.0 and SPSS 24.0 software. If the measured data fit a normal distribution, the values are presented as the mean ± standard deviation; for data that did not fit a normal distribution, the median (interquartile range) was used. Significant differences between groups were assessed by analysis of variance, the Kruskal–Wallis test, and the Mann–Whitney test. *P* < 0.05 was considered statistically significant.

## 4. Results

### 4.1. Identification of the Bioactive Compounds in QBD

From the seven active components of QBD, Guanghuoxiang (*Pogostemon cablin* (Blanco) Benth.), Gegeng (Radix Puerariae), Baizhu (*Atractylodes macrocephala* Koidz.), Rensheng (*Panax ginseng* C. A. Mey.), Fuling (*Poria cocos*(Schw.) Wolf.), Muxiang (Aucklandiae Radix), and Gancao (Licorice), 777 compounds were obtained from the Traditional Chinese Medicine Systems Pharmacology (TCMSP) database. Forty-six active chemical components of QBD were obtained with an OB% ≥ 30% (Glycyrrhiza is OB% ≥ 60%) and a DL ≥ 0.18, according to the screening standards. The properties of the compounds are shown in Supplementary Table 1.

### 4.2. Efficacy of QBD in Alleviating Diarrhea

Diarrhea was not observed in any of the rats in the control group. From 1 day after the ingestion of PEG, watery stools or diarrhea began to appear in the model group, *S. boulardii* group, and QBD group. Notably, rats in the model group exhibited obvious diarrhea, as determined by measuring fecal consistency and weight loss.

**Figure 1:**
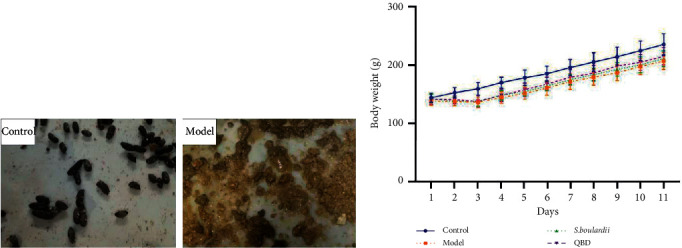
Efficacy of QBD in alleviating diarrhea. (a) State of feces. (b) Mean weight loss in the experimental groups.

On the first day of the experiment, the mean weights of rats in all groups were similar. During the study period, weight loss was not detected in the control group, but the weight of the rats in the remaining three groups decreased. From the second day of PEG treatment to the last day of the experiment, the mean weights of the model group, *S. boulardii* group, and QBD group were lower than that of the control group (*P* < 0.05). Pairwise comparisons between the model group and the *S. boulardii* and QBD groups showed that the *P* value was greater than 0.05, and there was no significant difference. However, the mean weights of the three groups were in the following order: QBD group > *S. boulardii* group > model group (see Figure 1).

### 4.3. Effect of QBD on the Colon Mucus Barrier

Alcian blue staining revealed that, in the model group, the mucus layer was extremely thin or even absent, only a few goblet cells secreted new mucus from the base, and most of the goblet cells did not secrete new mucus compared to the control group. Compared to the control group, the thickness of the mucus layer in the model group was significantly thinner (*P*=0.001).

In the *S. boulardii* group and QBD group, the mucus layer was incomplete but thicker and more continuous than that in the model group. Most goblet cells secreted new mucus from the base. Compared to the model group, the thickness of the mucus layer in the QBD group (*P*=0.06) and the *S. boulardii* group (*P*=0.22) was not statistically significant. However, the thickness of the mucus layer in the QBD group was not significantly different (*P*=0.08) from that in the blank control group. The results showed that both the *S. boulardii* group and QBD group showed reversal of PEG-induced mucus thinning compared with the model group.

**Figure 2:**
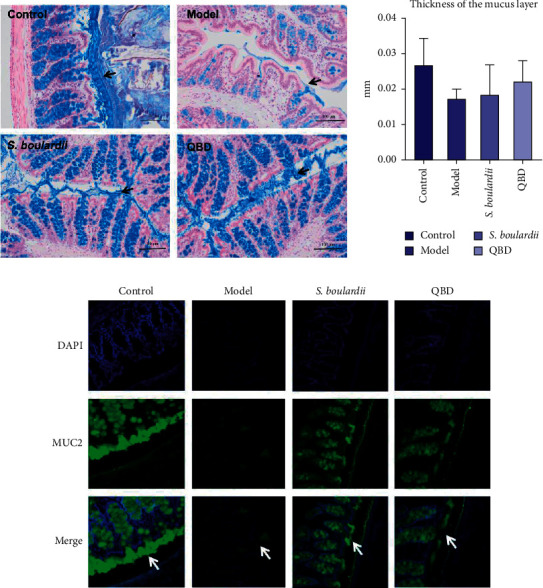
QBD alleviated colon mucus layer injury in diarrheal rats after treatment. (a) Alcian blue-stained colonic sections showing the mucus layer. The black arrow indicates the mucus layer. Scale bars, 100 *μ*m. (b) Colonic mucus layer measurements from Alcian blue-stained sections. Data are presented as the means, and error bars represent the SDs. (c) Expression of MUC2 proteins. White arrows indicate MUC2 proteins. ^#^*P* < 0.05 and ^##^*P* < 0.01 vs. the control group.

Immunofluorescence-stained sections revealed that the expression of MUC2 was extremely weak in the intestinal lumen and goblet cells compared to the control group. MUC2 had stronger fluorescence expression in the *S. boulardii* group and QBD group than in the model group, indicating that the layer thickness, the level of MUC2 expression, and the increase in MUC2 secretion by goblet cells were restored compared to those in the model group, consistent with the Alcian blue staining results (see Table 1 and Figure 2).

### 4.4. Effects of QBD Treatment on SCFAs in the Colon Contents

**Figure 3:**
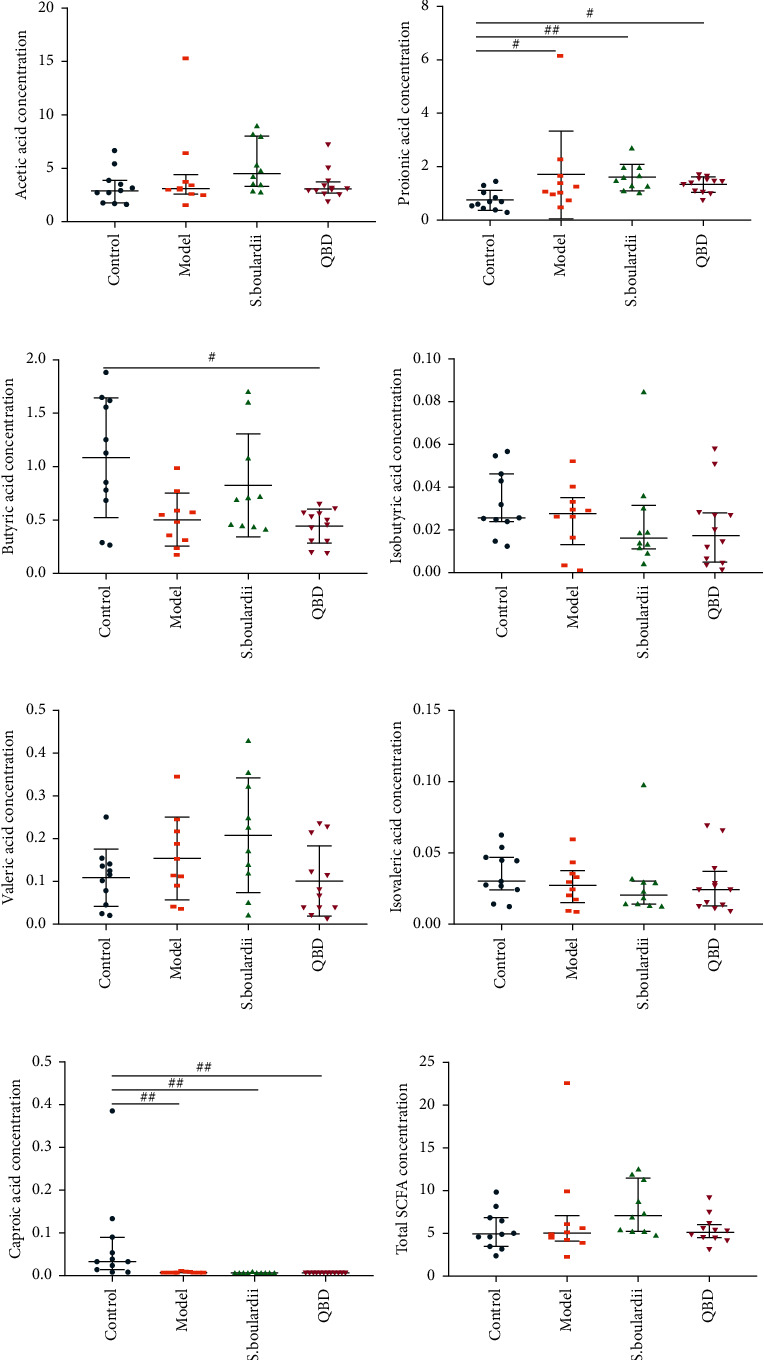
Concentrations of SCFAs in the colon of each group during the treatment. (a) Acetic acid. (b) Propionic acid. (c) Butyric acid. (d) Isobutyric acid. (e) Valeric acid. (f) Isovaleric acid. (g) Caproic acid. (h) Total short-chain fatty acids. ^#^*P* < 0.05 and ^##^*P* < 0.01 vs. control group.

Seven SCFAs were detected in the colon contents via GC-MS. The concentrations of total SCFAs, acetic acid, and propionic acid increased, and the propionic acid concentration (*P*=0.047) reached statistical significance in the model group compared to the control group. However, the level of butyric acid (*P*=0.02) in QBD group was significantly reduced compared to that in the control group. Although the difference was not statistically significant, the total levels of SCFAs, acetic acid, propionic acid, and butyric acid in the *S. boulardii* group were higher than those in the QBD group or the model group. These results showed that the effect of *S. boulardii* on SCFA production was better than that of QBD (see Figure 3).

### 4.5. Overall Structural Modulation of the Gut Microbiome during QBD Treatment

**Figure 4:**
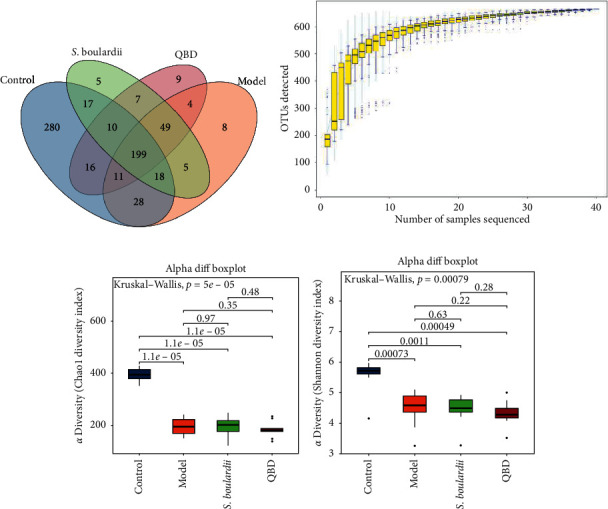
Overall structural modulation of the gut microbiome during QBD treatment. (a) Venn diagram indicating the differential numbers of operational taxonomic units (OTUs) in the control, model, *S. boulardii*, and QBD groups. (b) Species accumulation curves showing an adequate sampling depth for all samples. (c, d) Alpha diversity analysis of the gut microbiota in the experimental groups based on the Chao1 and Shannon indices. *e* indicates the natural logarithm.

The structural changes in the rat gut microbiota after QBD treatment were detected by 16S rDNA amplicon sequencing. A total of 1,420,548 reads (average of 35,514 sequences per sample) from 40 samples were delineated into 667 OTUs. The species accumulation curves showed an adequate sampling depth for all samples (see Figures 4(a) and 4(b)).

Alpha diversity indices (Chao1 and Shannon) reflect community richness and uniformity. The highest community richness and uniformity were observed in the control group. Compared with the control group, the Chao1 index and Shannon index were significantly lower in the model group. However, the differences in the Shannon index among the model, *S. boulardii*, and QBD groups were not significant (see Figures 4(c) and 4(d)).

**Figure 5:**
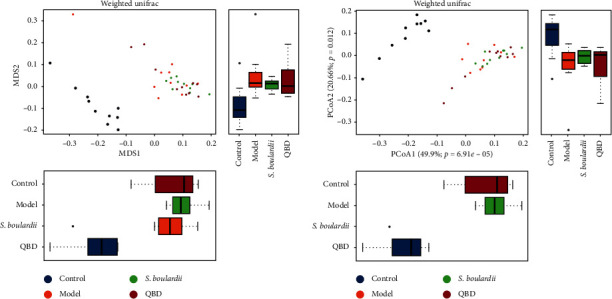
(a) Nonmetric multidimensional scaling (NMDS) and (b) principal coordinate analysis (PCoA) scores based on weighted Unifrac metrics indicating different beta diversity of gut microbiota among the experimental groups.

Beta diversity analysis includes nonmetric multidimensional scaling (NMDS) and principal co-ordinates analysis (PCoA). The NMDS and PCoA results showed that the distance between the control group and the other three groups was large. In summary, PEG treatment reduced the species diversity and structure of the gut microbiota in rats, while *S. boulardii* and QBD treatment did not significantly change the species diversity and structure of the gut microbiota (see Figures 5(a) and 5(b)).

### 4.6. Key Phylotypes of the Gut Microbiota Modulated by the QBD

The composition of the gut microbiota at different taxonomic levels was analyzed to determine which types of bacteria were affected by *S. boulardii* and QBD intake.

**Figure 6:**
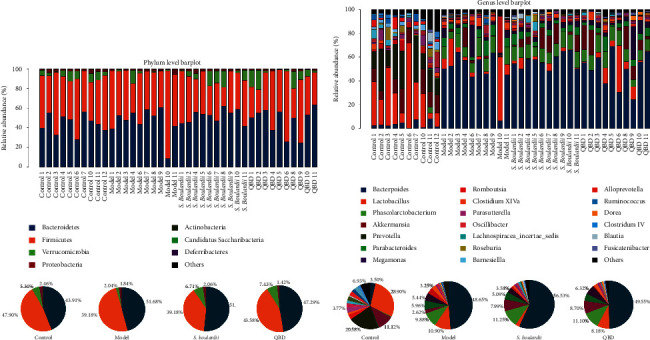
(a) The histogram shows the relative abundance at the phylum level in the 40 samples, and the pie charts show the corresponding microbiota community structures in the experimental group. (b) The histogram shows the relative abundance at the genus level in the 40 samples, and the pie charts show the corresponding microbiota community structures in the experimental group.

Eight phyla were found in all of the samples, among which the most abundant were Bacteroidetes, Firmicutes, Verrucomicrobia, and Proteobacteria. At the genus level, twenty-two genus-dominant genera were identified in the experimental groups, among which the most abundant were *Bacteroides, Lactobacillus, Phascolarctobacterium,* and *Akkermansia* (see Figures 6(a) and 6(b)).

**Figure 7:**
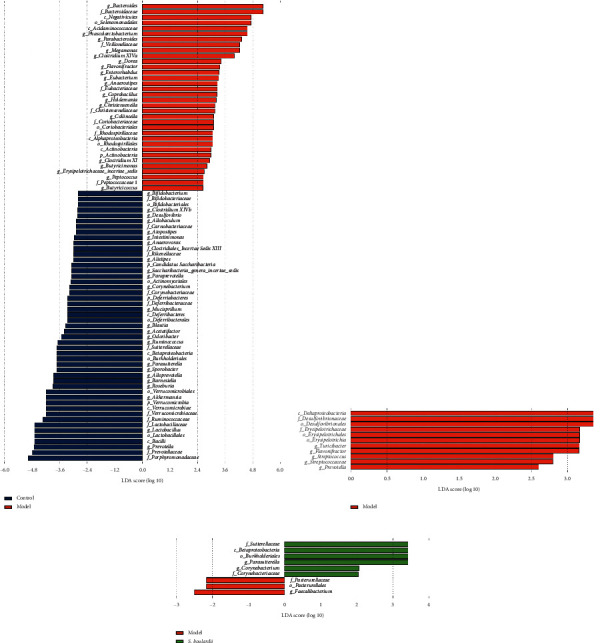
(a) LEfSE comparison of gut microbiota between the control and model groups. (b) LEfSE comparison of gut microbiota between the model and QBD groups. (c) LEfSE comparison of gut microbiota between the model and *S. boulardii* groups.

LEfSe analysis is an analysis method that uses linear discriminant analysis (LDA) to estimate the impact of the abundance of each species on diversity and identify communities or species that show significant differences in sample partitioning. A logarithmic LDA score cutoff of 4.0 was used to identify important taxonomic differences (see Figures 7(a)–7(c)).

In summary, compared to the control group, the abundances of Verrucomicrobia (*P*=0.043), *Akkermansia* (*P*=0.043), *Lactobacillus* (*P*=0.035), and *Prevotella* (*P*=0.0001) were significantly decreased in the model group. In addition, the abundances of *Bacteroides* (*P*=0), *Phascolarctobacterium* (*P*=0.0002), *Parabacteroides* (*P*=0.0003), and *Clostridium XlVa* (*P*=0.011) were significantly increased in the model group compared with the control group. Furthermore, the relative abundances of Verrucomicrobia in the control group, model group, *S. boulardii* group, and QBD treatment group were 5.30%, 2.04%, 6.71%, and 7.43%, respectively. Moreover, the relative abundances of *Akkermansia* accounted for 11.12%, 2.62%, 7.99%, and 8.70% in the control group, model group, *S. boulardii* group, and QBD treatment group, and QBD is higher than *S. boulardii* treatment. Diarrhea reduced the relative abundances of Verrucomicrobia and *Akkermansia.* Although the relative abundances of Verrucomicrobia and *Akkermansia* among the three groups did not reach statistical significance, the *S. boulardii* and QBD treatments enhanced the increasing tendencies of Verrucomicrobia and *Akkermansia*. Moreover, the abundances of *Turicibacter* (*P*=0.037) and *Flavonifractor* (*P*=0.043) were significantly increased in the model group compared with the QBD group. Compared to the model group, the abundance of *Parasutterella* (*P*=0.043) was significantly increased in the *S. boulardii* group.

### 4.7. Spearman Correlation Analysis of the Gut Microbiota and SCFAs

**Figure 8:**
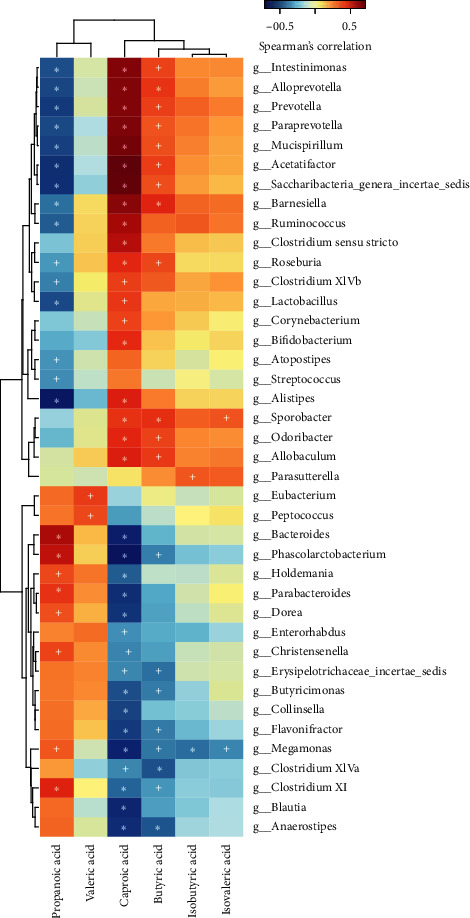
Heat map of the Spearman correlation analysis between the gut microbiota and SCFAs. ^+^*P* < 0.05; ^*∗*^*P* < 0.01.

The different bacterial genera produced by the 16S rDNA amplicon sequencing analysis and the SCFA concentration were determined by Spearman correlation analysis. The results were as follows. There was no correlation between acetic acid and any of the different bacterial genera (*P* > 0.05). Propionic acid was positively correlated with *Bacteroides*, *Parabacteroides*, and *Phascolarctobacterium* but negatively correlated with *Prevotella* and *Lactobacillus*. Butyric acid was positively correlated with *Prevotella* and negatively correlated with *Clostridium XlVa*. Caproic acid was positively correlated with *Prevotella* and *Lactobacillus* and negatively correlated with *Bacteroides*, *Phascolarctobacterium*, and *Flavonifractor* (see Figure 8 and Supplementary Table 2).

## 5. Discussion

The balance of the gut microbiota is closely related to diarrhea. Imbalance between diarrhea and the gut microbiota is a result of mutual cause and effect. The diversity of the gut microbiota of healthy people is higher than that of patients with diarrhea under normal conditions [46]. Although the therapeutic effect of QBD on diarrhea has been confirmed in clinical research, the general mechanism remains unclear. Based on these observations, in this study, 16S rDNA high-throughput sequencing was used to explore the regulatory effect of QBD on the gut microbiota in diarrheal juvenile rats.

In this study, through analysis of sequencing data, a significant difference in the species diversity and structures of the gut microbiota in rats was found between the control group and the model group, while the *S. boulardii* and QBD treatments did not significantly change the species diversity and structures of the gut microbiota, which was consistent with the study of Long et al. [27]. These results may be due to the continuous effect of PEG treatment via drinking water; the *S. boulardii* and QBD treatments once daily were not enough to reverse the changes in the species diversity and structures of the gut microbiota caused by PEG 3350.

Analysis of the differences in the intestinal flora showed the following:

1. Compared with the control group, the abundances of *Bacteroides, Parabacteroides, Phascolarctobacterium,* and *Clostridium XIVa* were significantly increased in the model group, and the abundances of Verrucomicrobia*, Lactobacillus, Akkermansia,* and *Prevotella* were significantly reduced in the model group. Specifically, *Clostridium XIVa* is closely related to protein fermentation and an increased risk of diarrhea [47]. Pop et al. [48] also observed that the abundance of *Prevotella* in children with diarrhea in low-income countries was decreased. Moreover, the abundance of *Akkermansia* in the feces of pigs suffering from epidemic diarrhea was significantly decreased. The abundance of *Lactobacillus* in patients with diarrheal irritable bowel syndrome was significantly decreased, and *Lactobacillus* treatment significantly improved the symptoms [49]. In addition, the abundances of *Lactobacillus, Prevotella, Akkermansia*, and Verrucomicrobia in the feces of *E. coli* O101 diarrhea model rats were significantly reduced. Our results are consistent with these findings.
2. In summary, *Clostridium XIVa* may have the effect of exacerbating diarrhea, while Verrucomicrobia*, Lactobacillus, Akkermansia, and Prevotella* may be beneficial bacteria that have a protective effect against diarrhea; therefore, the imbalance between the increased abundance of *Clostridium XIVa* and decreased abundance of Verrucomicrobia*, Lactobacillus, Akkermansia,* and *Prevotella* may be related to the mechanism underlying diarrhea.
3. The concentrations of total SCFAs, acetic acid, and propionic acid were increased, and the propionic acid concentration reached statistical significance in the model group compared to the control group. These findings are similar to those of Whelan et al., who found that the concentrations of fecal total SCFAs, acetic acid, and butyrate increased in patients with diarrhea who were receiving enteral feeding [50]. Moreover, Li et al. also observed that the concentration of propionic acid in the ascending colon of young pigs with rotavirus diarrhea was increased [51]. In this experiment, the abundances of propionic acid-producing bacteria, *Bacteroides, Parabacteroides,* and *Phascolarctobacterium* in the three groups using PEG 3350 were significantly increased. The results of Spearman's correlation analysis also confirmed that *Bacteroides, Parabacteroides,* and *Phascolarctobacterium* were positively correlated with propionic acid. This may be the reason for the increased concentration of propionic acid in diarrhea samples. A mechanistic explanation may be that the faster transit time in the small intestine during diarrhea leads to malabsorption of carbohydrates over this short period of time, which increases the level of substrate for colonic fermentation, promoting decomposition by these flora, and leading to increased SCFA production [52, 53].
4. QBD treatment enhanced the increasing tendencies of the abundances of Verrucomicrobia and *Akkermansia*, whereas those of *Turicibacter* and *Flavonifractor* were significantly reduced, compared to the model group. To date, there have been few studies on *Turicibacter* and *Flavonifractor*, which are mostly negatively related to health [54–56]. Importantly, accumulating studies have indicated that Verrucomicrobia and *Akkermansia* are closely related to diarrhea and the intestinal mucus barrier. Therefore, we hypothesized that QBD treatment modulates the gut microbiota and repairs the mucus barrier in juvenile diarrheal rats by the following mechanism: (1) QBD treatment prompts an increase in Verrucomicrobia abundance and thereby improves the intestinal barrier [57]. (2) QBD treatment increases the abundance of *Akkermansia*, the most representative bacteria in the phylum Verrucomicrobia. *Akkermansia* and mucin glycoside hydrolase expression help maintain the thickness of the mucosal layer during PEG-induced diarrhea, which suggests a microbiota-driven feedback mechanism aiding mucus resilience [3].
5. Importantly, the mucus barrier and its most important protein, MUC2, protect the intestines and prevent diarrhea. Xu et al. found that MUC2 is an important protein for the prevention and treatment of rotavirus infection and diarrhea, acting by protecting the epithelial barrier and resisting intestinal permeability [58]. Wang et al. also observed that increasing the content of MUC2 in the ileum of rats with diarrhea can enhance intestinal barrier defense and prevent diarrhea [59]. Interestingly, TCM herbs (fermented Rhizoma Atractylodis Macrocephalae) increase the proportion of *Akkermansia* and simultaneously have a beneficial effect on host metabolism [60]. Zhang et al. also found that Sijunzi decoction contained in QBD increased the expression of MUC2 and improved the state of spleen deficiency in rats [61]. Sun et al. previously showed that QBD regulated the imbalance of the gut microbiota and promoted the repair of intestinal mucosal damage in diarrheal mice [62]. Our results are consistent with this finding.
6. In summary, based on the results showing that the mucus layer thickness and MUC2 expression in the QBD group were greater than those in the model group, it is suggested that QBD may regulate the intestinal flora, such as by increasing the abundances of Verrucomicrobia and *Akkermansia*, to repair the mucus barrier, protect the intestines, and treat diarrhea.
7. The abundance of *Parasutterella* in the *S. boulardii* group was significantly higher than that in the model group. Chen et al. observed that pectin increased the abundances of *Bacteroides* and *Akkermansia* and significantly increased the abundances of *Parasutterella*, which may have contributed to increasing the concentrations of acetic acid, propionic acid, and butyric acid in the cecum [63]. Li et al. reported that reductions in the abundance of *Lactobacillus, Parasutterella,* and *Desulfovibrio* and the levels of acetic acid, propionic acid, and butyric acid were consistent after azithromycin treatment in mice [64]. The results of these two studies are consistent with our findings regarding the change trends of some gut microbes and SCFAs, suggesting that the abundance of *Parasutterella* is positively correlated with the levels of acetic acid, propionic acid, and butyric acid.

The GC-MS results showed that although the difference was not statistically significant, the total levels of SCFAs, acetic acid, propionic acid, and butyric acid in the *S. boulardii* group were higher than those in the model group. Pipau et al. found that *S. boulardii* alleviated diarrhea by increasing the concentrations of acetic acid, propionic acid, and butyric acid [65].

**Figure 9:**
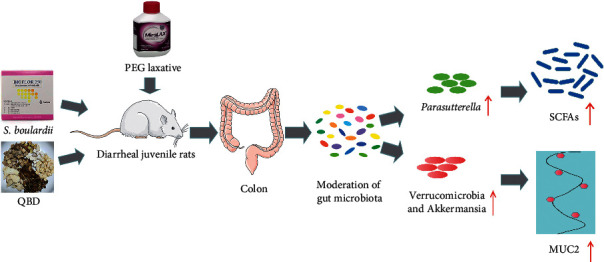
Possible mechanism underlining *S. boulardii* and QBD treatment of diarrhea in juvenile rats (note: the rat and colon images are from https://smart.servier.com/).

Based on these findings, we speculate that *S. boulardii* can increase the abundance of *Parasutterella*, resulting in an increase in the levels of acetic acid, propionic acid, and butyric acid, consequently leading to alleviation of diarrhea (see Figure 9).

## 6. Conclusion

QBD has a tendency to improve the weight gain of juvenile rats with diarrhea. QBD has a stronger ability to repair the mucus barrier than *S. boulardii*, but its ability to improve SCFAs is not as good as that of *S. boulardii*. It is suggested that *S. boulardii* may be able to treat diarrhea by increasing the abundance of *Parasutterella* significantly to increase SCFA production, and the mechanism of action of QBD in the treatment of juvenile rats with diarrhea likely involves gut microbiota-mediated repair of the mucus barrier.

## Figures and Tables

**Table 1:** Colonic mucus thickness (mean ± SD, *n* = 39).

Group	Thickness of the mucus layer (mm)
Control group	0.028 ± 0.008
Model group	0.017 ± 0.003^##^
*S. boulardii* group	0.021 ± 0.005^#^
QBD group	0.022 ± 0.006
*F*	4.68
*P*	0.006

^#^ *P* < 0.05 and ^##^*P* < 0.01 vs. control group. SD, standard deviation.

## Data Availability

The data used to support the findings of this study are available from the corresponding author upon request.

## Supplementary Materials

Supplementary Materials Supplementary Table 1: the active ingredients of the seven components of QBD. Supplementary Table 2: Spearman correlation analysis between the gut microbiota and SCFAs.
