# Supplementary material for: Qiweibaizhu Decoction Treats Diarrheal Juvenile Rats by Modulating the Gut Microbiota, Short-Chain Fatty Acids, and the Mucus Barrier
Source: Evid Based Complement Alternat Med. 2021 Jan 17;2021:8873294. doi: 10.1155/2021/8873294 (PMC7834800; doi:10.1155/2021/8873294)
Supplement: Supplementary Materials — Supplementary Table 1: the active ingredients of the seven components of QBD. Supplementary Table 2: Spearman correlation analysis between the gut microbiota and SCFAs. [file 8873294.f1.doc]

Supplementary Table 1. The active ingredients of the seven components of QBD.

| Mol ID | Herb name | Molecule Name | OB (%) | DL | Structure |
| --- | --- | --- | --- | --- | --- |
| MOL002879 | (Guanghuoxiang) Pogostemon Cablin (Blanco) Benth. | [Diop](https://tcmspw.com/molecule.php?qn=2879) | 43.59 | 0.39 | 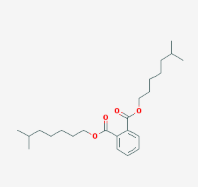 |
| MOL005573 | (Guanghuoxiang) Pogostemon Cablin (Blanco) Benth. | [Genkwanin](https://tcmspw.com/molecule.php?qn=5573) | 37.13 | 0.24 | 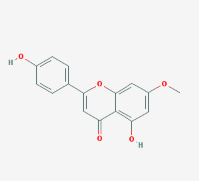 |
| MOL005911 | (Guanghuoxiang) Pogostemon Cablin (Blanco) Benth. | [5-Hydroxy-7,4'-dimethoxyflavanon](https://tcmspw.com/molecule.php?qn=5911) | 51.54 | 0.27 | 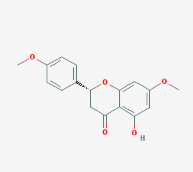 |
| MOL005916 | (Guanghuoxiang) Pogostemon Cablin (Blanco) Benth. | [Irisolidone](https://tcmspw.com/molecule.php?qn=5916) | 37.78 | 0.30 | 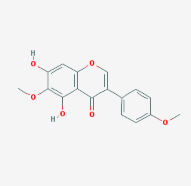 |
| MOL000392 | (Gegen) Radix Puerariae | Formononetin | 69.67 | 0.21 | 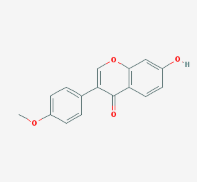 |
| MOL000358 | (Gegen) Radix Puerariae | Beta-sitosterol | 36.91 | 0.75 | 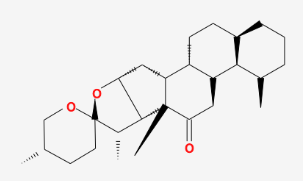 |
| MOL000028 | (Baizhu) Atractylodes Macrocephala Koidz. | α-Amyrin | 39.51 | 0.76 | 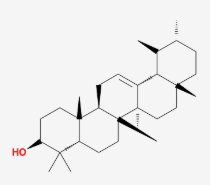 |
| MOL000049 | (Baizhu) Atractylodes Macrocephala Koidz | [3β-acetoxyatractylone](https://tcmspw.com/molecule.php?qn=49) | 54.07 | 0.22 | 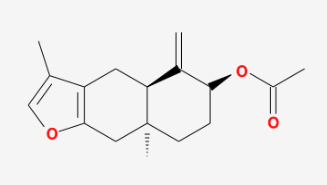 |
| MOL000072 | (Baizhu) Atractylodes Macrocephala Koidz | 8β-ethoxyatractylenolide Ⅲ | 35.95 | 0.21 | 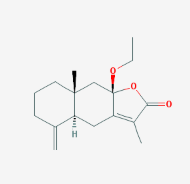 |
| MOL000022 | (Baizhu) Atractylodes Macrocephala Koidz | 14-acetyl-12-senecioyl-2E,8Z,10E-atractylentriol | 63.37 | 0.30 | 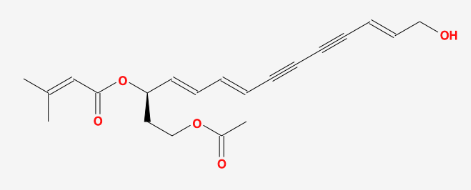 |
| MOL002879 | (Renshen) Panax Ginseng C. A. Mey. | Diop | 43.59 | 0.39 | 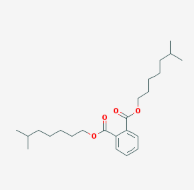 |
| MOL000449 | (Renshen) Panax Ginseng C. A. Mey. | Stigmasterol | 43.83 | 0.76 | 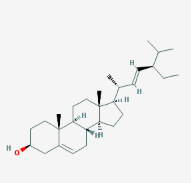 |
| MOL000358 | (Renshen) Panax Ginseng C. A. Mey. | Beta-sitosterol | 36.91 | 0.75 | 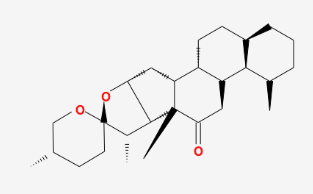 |
| MOL003648 | (Renshen) Panax Ginseng C. A. Mey. | Inermin | 65.83 | 0.54 | 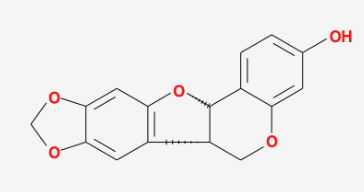 |
| MOL000422 | (Renshen) Panax Ginseng C. A. Mey. | Kaempferol | 41.88 | 0.24 | 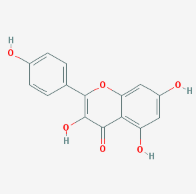 |
| MOL004492 | (Renshen) Panax Ginseng C. A. Mey. | [Chrysanthemaxanthin](https://tcmspw.com/molecule.php?qn=4492) | 38.72 | 0.58 | 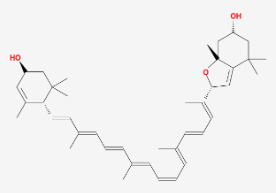 |
| MOL005308 | (Renshen) Panax Ginseng C. A. Mey. | [Aposiopolamine](https://tcmspw.com/molecule.php?qn=5308) | 66.65 | 0.22 | 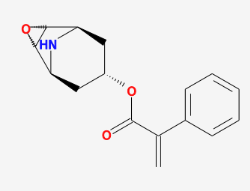 |
| MOL005356 | (Renshen) Panax Ginseng C. A. Mey. | [Girinimbin](https://tcmspw.com/molecule.php?qn=5356) | 61.22 | 0.31 | 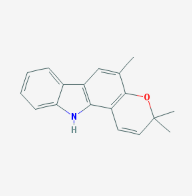 |
| MOL005314 | (Renshen) Panax Ginseng C. A. Mey. | [Celabenzine](https://tcmspw.com/molecule.php?qn=5314) | 101.88 | 0.49 | 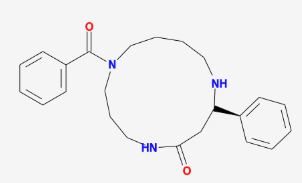 |
| MOL005399 | (Renshen) Panax Ginseng C. A. Mey. | [Alexandrin_qt](https://tcmspw.com/molecule.php?qn=5399) | 36.91 | 0.75 | 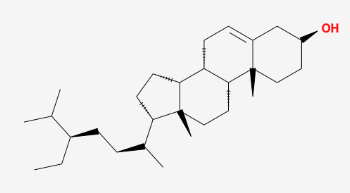 |
| MOL000787 | (Renshen) Panax Ginseng C. A. Mey. | Fumarine | 59.26 | 0.83 | 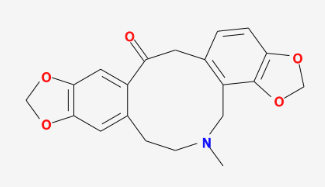 |
| MOL005401 | (Renshen) Panax Ginseng C. A. Mey. | Ginsenoside Rg5_qt | 39.56 | 0.79 | 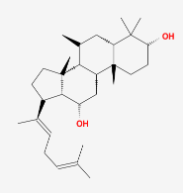 |
| MOL005318 | (Renshen) Panax Ginseng C. A. Mey. | Dianthramine | 40.45 | 0.20 | 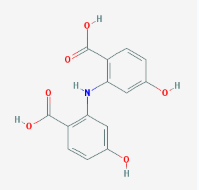 |
| MOL005317 | (Renshen) Panax Ginseng C. A. Mey. | Deoxyharringtonine | 39.27 | 0.81 | 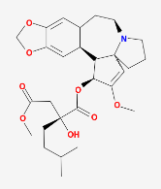 |
| MOL005320 | (Renshen) Panax Ginseng C. A. Mey. | [Arachidonate](https://tcmspw.com/molecule.php?qn=5320) | 45.57 | 0.20 | 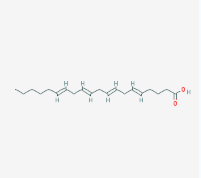 |
| MOL005321 | (Renshen) Panax Ginseng C. A. Mey. | Frutinone A | 65.90 | 0.34 | 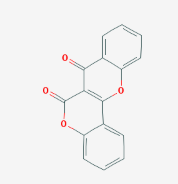 |
| MOL005344 | (Renshen) Panax Ginseng C. A. Mey. | [Ginsenoside rh2](https://tcmspw.com/molecule.php?qn=5344) | 36.32 | 0.56 | 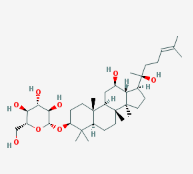 |
| MOL005348 | (Renshen) Panax Ginseng C. A. Mey. | Ginsenoside-Rh4_qt | 31.11 | 0.78 | 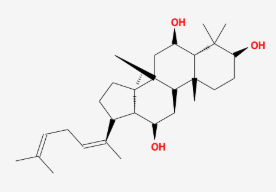 |
| MOL005357 | (Renshen) Panax Ginseng C. A. Mey. | Gomisin B | 31.99 | 0.83 | 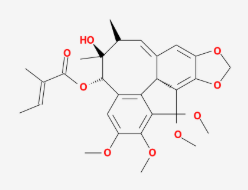 |
| MOL005360 | (Renshen) Panax Ginseng C. A. Mey. | Malkangunin | 57.71 | 0.63 | 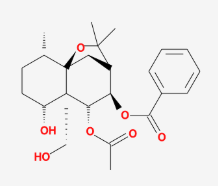 |
| MOL005376 | (Renshen) Panax Ginseng C. A. Mey. | Panaxadiol | 33.09 | 0.79 | 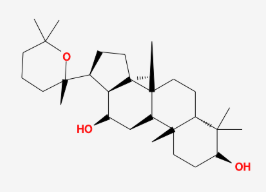 |
| MOL005384 | (Renshen) Panax Ginseng C. A. Mey. | Suchilactone | 57.52 | 0.56 | 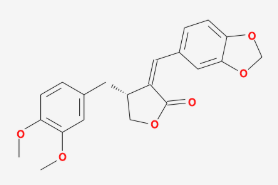 |
| MOL005399 | (Renshen) Panax Ginseng C. A. Mey. | Alexandrin_qt | 36.91 | 0.75 | 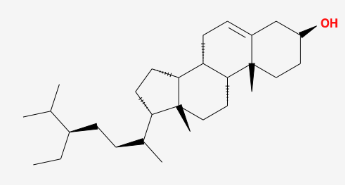 |
| MOL000275 | (Fuling) Poria Cocos(Schw.) Wolf. | [Trametenolic acid](https://tcmspw.com/molecule.php?qn=275) | 38.71 | 0.80 | 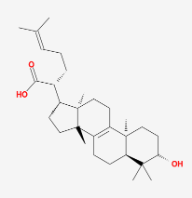 |
| MOL000283 | (Fuling) Poria Cocos(Schw.) Wolf. | [Ergosterol peroxide](https://tcmspw.com/molecule.php?qn=283) | 40.36 | 0.81 | 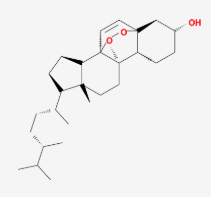 |
| MOL000279 | (Fuling) Poria Cocos(Schw.) Wolf. | [Cerevisterol](https://tcmspw.com/molecule.php?qn=279) | 37.96 | 0.77 | 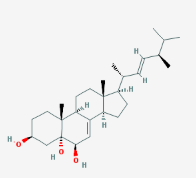 |
| MOL000296 | (Fuling) Poria Cocos(Schw.) Wolf. | [Hederagenin](https://tcmspw.com/molecule.php?qn=296) | 36.91 | 0.75 | 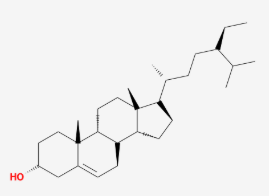 |
| MOL010813 | (Muxiang) Aucklandiae Radix | Benzo[a]carbazole | 35.22 | 0.22 | 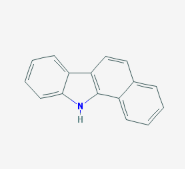 |
| MOL010839 | (Muxiang) Aucklandiae Radix | Lappadilactone | 38.56 | 0.73 | 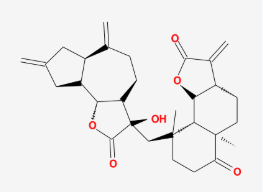 |
| MOL000359 | (Muxiang) Aucklandiae Radix | Sitosterol | 36.91 | 0.75 | 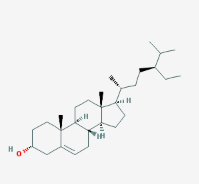 |
| MOL010828 | (Muxiang) Aucklandiae Radix | [Cynaropicrin](https://tcmspw.com/molecule.php?qn=10828) | 67.50 | 0.38 | 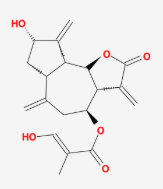 |
| MOL000211 | (Muxiang) Aucklandiae Radix | [Mairin](https://tcmspw.com/molecule.php?qn=211) | 55.38 | 0.78 | 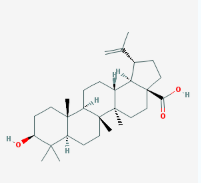 |
| MOL000449 | (Muxiang) Aucklandiae Radix | Stigmasterol | 43.83 | 0.76 | 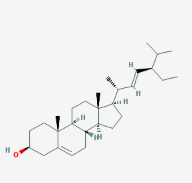 |
| MOL002311 | (Gancao) licorice | [Glycyrol](https://tcmspw.com/molecule.php?qn=2311) | 90.78 | 0.67 | 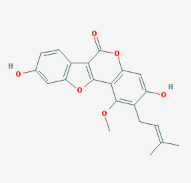 |
| MOL004891 | (Gancao) licorice | [Shinpterocarpin](https://tcmspw.com/molecule.php?qn=4891) | 80.30 | 0.73 | 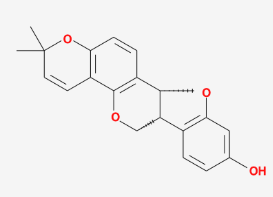 |
| MOL004990 | (Gancao) licorice | [7,2',4'-trihydroxy－5-methoxy-3－arylcoumarin](https://tcmspw.com/molecule.php?qn=4990) | 83.71 | 0.27 | 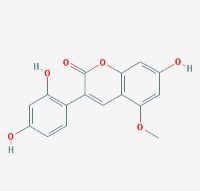 |
| MOL004841 | (Gancao) licorice | [Licochalcone B](https://tcmspw.com/molecule.php?qn=4841) | 76.76 | 0.19 | 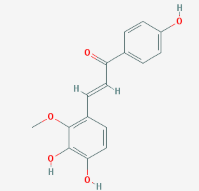 |
| MOL004904 | (Gancao) licorice | [Licopyranocoumarin](https://tcmspw.com/molecule.php?qn=4904) | 80.36 | 0.65 | 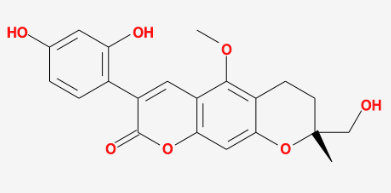 |

Note: the structure images are from the Traditional Chinese Medicine Systems Pharmacology (TCMSP) database.

Supplementary Table 2. Spearman correlation analysis between the gut microbiota and SCFAs

|  | Propionic acid | Isobutyric acid | Butyric acid | Isovaleric acid | Valeric acid | Caproic acid | *P* value |
| --- | --- | --- | --- | --- | --- | --- | --- |
| *Bacteroides* | 0.350356205 | 0.000111773 | 0.690684167 | 0.11088403 | 0.69887522 | 0.384062287 | 7.42E-06 |
| *Clostridium* *XlVa* | 0.175607705 | 0.239287151 | 0.209724922 | 0.002082285 | 0.242821997 | 0.254159854 | 0.027845415 |
| *Flavonifractor* | 0.620811452 | 0.088478969 | 0.132237277 | 0.017493952 | 0.299752947 | 0.47470232 | 0.000334611 |
| *Lactobacillus* | 0.984800486 | 0.001333919 | 0.337023329 | 0.311321067 | 0.390203804 | 0.853221407 | 0.011243037 |
| *Parabacteroides* | 0.941496818 | 0.009033153 | 0.658394007 | 0.06972638 | 0.94733853 | 0.20474084 | 8.93E-05 |
| *Parasutterella* | 0.253220945 | 0.742185833 | 0.048688276 | 0.199370007 | 0.057821195 | 0.615160449 | 0.753103264 |
| *Phascolarctobacterium* | 0.299511139 | 0.000296063 | 0.1685744 | 0.020359114 | 0.245611764 | 0.560938287 | 9.61E-07 |
| *Prevotella* | 0.265159121 | 0.000360248 | 0.064956615 | 0.019547992 | 0.141959399 | 0.731458568 | 7.14E-06 |

E indicates the natural logarithm.
